# Supplementary material for: Exploring EFL teachers’ beliefs and practices of formative assessment in Chinese context
Source: PLoS One. 2025 Sep 29;20(9):e0333678. doi: 10.1371/journal.pone.0333678 (PMC12478959; doi:10.1371/journal.pone.0333678)
Supplement: S1 Appendix — (DOCX) [file pone.0333678.s001.docx]

**Appendix A: Questionnaire**

1. Your gender is

A. Male

B. Female

2. Your teaching experience is

A. 1-5 years

B. 6-10 years

C. 11-15 years

D. ≥16 Years

3. Your academic title is

A. Lecturer and below

B. Associate professor

C. Full professor

4. How important do you consider formative assessment in your teaching?

A. Not important at all

B. Slightly important

C. Neutral

D. Fairly important

E. Very important

5. Which of the following best describes formative assessment in your opinion? (Select all that apply)

A. It is a theory that promotes student learning through continuous feedback.

B. It involves informal methods such as classroom discussions and observations to monitor learning progress and guide instructional adjustments.

C. It consists of regular quizzes or assignments to understand students' learning status and improve teaching.

D. It involves student self-assessment and peer assessment to enhance self-reflection skills and learning outcomes.

E. Uncertain

6. What aspects do you usually assess with formative assessment in your classroom? (Select all that apply)

A. Language skills (listening, speaking, reading, writing)

B. Participation and collaboration abilities

C. Learning attitude and effort level

D. Mastery of knowledge points

E. Others, please specify __________

7. What do you believe are the main purposes of using formative assessment? (Select all that apply)

A. Improving Classroom Engagement

B. Enhancing learning Motivation

C. Improving Teacher-Student Relationship

D. Boosting students’ Confidence

E. Improving Learning habits

F. Enhancing Teamwork

G. Others, please specify __________

8. What do you base your determination of the frequency and number of formative assessments on?

A. Teaching experience

B. School regulations

C. Course teaching objectives

D. Others, please specify __________

9. How often do you implement formative assessment in your English classes each semester?

A. Less than 5 times

B. 5-10 times

C. 11-20 times

D. More than 20 times

10. Which formative assessment strategies do you use most often in your teaching? (Select all that apply)

A. Classroom questioning

B. Individual assignments

C. Group projects

D. Periodic quizzes/tests

E. Student self-assessment

F. Peer assessment

G. Others, please specify __________

11. Which tools or technologies do you commonly use when implementing formative assessments? (Select all that apply)

A. Textbook-publisher supported teaching platforms

B. Commercial teaching platforms

C. Social media like QQ groups, WeChat groups

D. Paper materials

E. Language learning apps or mini-programs

F. Others, please specify __________

12. For the formative assessment results in reality, you mainly use them for (Select all that apply)

A. To refine teaching methods

B. To calculate students’ course average grades

C. To promote classroom participation

D. To help students understand their learning progress

E. To meet the institute’s requirements

F. To encourage student teamwork

G. Others, please specify __________

13. What are the main factors that influence the effectiveness of your implementation of formative assessments in teaching? (Select all that apply)

A. Insufficient time

B. Inadequate technical support

C. Low student engagement

D. Obscure evaluation criteria

E. Heavy evaluation workload

F. Lack of training

G. Lack of objectivity and fairness in self- and peer-assessment

H. Others, please specify __________

14. Regarding your current practice of formative assessments, what experiences can you share? What ideas do you have for improvement?
