# Supplementary material for: Exploring EFL teachers’ beliefs and practices of formative assessment in Chinese context
Source: PLoS One. 2025 Sep 29;20(9):e0333678. doi: 10.1371/journal.pone.0333678 (PMC12478959; doi:10.1371/journal.pone.0333678)
Supplement: S2 Appendix — (DOCX) [file pone.0333678.s002.docx]

**Appendix B: Interview Questions**

1. What do you think is formative assessment?

2. How do you view formative assessment in English teaching?

3. In English teaching, how do you implement formative assessment?

4. How do you use the formative assessment results?

5. What are the main difficulties you encounter when implementing formative assessment?
