# Supplementary material for: Exploring EFL teachers’ beliefs and practices of formative assessment in Chinese context
Source: PLoS One. 2025 Sep 29;20(9):e0333678. doi: 10.1371/journal.pone.0333678 (PMC12478959; doi:10.1371/journal.pone.0333678)
Supplement: Attachment — Submitted filename: Response to Reviewers.docx [file pone.0333678.s005.docx]

Dear Editor and Reviewers,

It is our pleasure to send you the revised version of our manuscript following the comments from the editor and two reviewers. We have tried our best to take into consideration the valuable suggestions and comments from the three, and then turn them into action to improve the quality of our manuscript. We thank them for their time and hard work in making our paper a better one.

Following your instructions,we have carefully addressed each point raised by the editor and reviewers. The following tables summarize the actions we have taken in response to the editor’s and reviewers’ comments together with the locations where you can find those changes in the revised version.

We hope these summairies have shown to you our best effort to improve our manuscript for potential publication in Plos One journal. We are grateful for your consideration. However, if there is anything we can do more to improve this revised manuscript, please do not hesitate to let us know.

We are looking forward to hearing your final decision on our manuscript from you. Thank you very much.

Sincerely,

Wanrong Lei

Zhibin Lei

| **Editor’s comments** | Response | Location (on the clean version) |
| --- | --- | --- |
| 1. Please ensure that your manuscript meets PLOS ONE’s style requirements, including those for file naming. | We have changed accordingly:  (1)Double-spaced the text | Whole text |
|  | (2)Included page and line numbers | Whole text |
|  | (3)reformatted references and in-text citations according to the “Vancouver” style | Within the text and “References” |
| 2.You indicated that ethical approval was not necessary for your study. Could you please provide further details on why your study is exempt from the need for approval and confirmation from your institutional review board or research ethics committee? | We have changed accordingly.  Provided a “Statement of Ethics Review Exemption” with detailed justification. | Attached as “Other” in supplementary materials. |
| 3. In the ethics statement in the Methods, you have specified that verbal consent was obtained. Please provide additional details regarding how this consent was documented and witnessed, and state whether this was approved by the IRB. | We have changed accordingly.  (1) Provided detailed documentation and witnessing of verbal consent in “Methods”;  (2)Added a new “ethical considerations” section. | Section 3.1 Participants (1^st^ Para.)  Section 3.5 Ethical Considerations |
| 4. We note that the grant information you provided in the “Funding Information” and “Financial Disclosure” sections do not match.please ensure that you provide the correct grant numbers for the awards you received for your study in the “Funding Information” section. | We have examined accordingly.  Confirmed that the grant numbers and funders listed in the “Funding Information” section are correct.  (We acknowledge and apoligize that the “Financial Disclosure” section was not clearly stated.) | “Funding information”. |
| 5. We note that your Data Availability Statement is currently as follows: [All relevant data are within the manuscript and its Supporting Information files.]  Please confirm at this time whether or not your submission contains all raw data required to replicate the results of your study. | We have changed accordingly.  Confirmed inclusion of minimal data set required for replication. | Attached as “Minimal data set” in Supporting Information. |
| 6.Please include captions for your Supporting Information files at the end of your manuscript, and update any in-text citations to match accordingly. | We have changed accordingly.  (1) Captions for all Supporting Information files (S1,S2,S3,S4)have been added at the end of the manuscript.  (2) Confirmed in-text citation for S3.  (3) No citations needed for S1, S2, and S4. |  |
| 7. Please review your reference list to ensure that it is complete and correct. | The reference list has been reviewed and found to be complete and accurate. | “References” section |

| **Additional Editor Comments** |  |  |
| --- | --- | --- |
| Issues to be addressed | Response | Location(on the clean version) |
| 1. Generalizability of the Sample - A reviewer mentioned that your data is confined to a specific geographical region. You should address this limitation in your ‘limitations’ section by arguing that your study can be expanded in future research through broader sampling and focused FA practices. Additionally, discuss these points in the future implications section. Furthermore, revise your ‘Discussion’ and ‘Conclusion’ sections accordingly. | (1)added a “Limitations” section addressing geographical limitation;  (2)discussed expansion opportunities in future implications of “limitation” section;  (3)Revised discussion and conclusion sections. | Section 6 Limitations  (p. 44-45)  Section 6 Limitations (p.45, Line 873-875)  Section 5.3 (p.42 Line 820-823);  Section 7 Conclusion |
| 2. Methodological Limitations  - According to the nature of your study, self-reported data may introduce bias, which could be clarified by expanding your methodology and focusing on future plans. This should also be addressed in your discussion while citing previous studies in the context of educational research. | (1) Acknowledged and addressed self-reporting bias in “Discussion” sectionin with relevant citations;  (2) Elaborated on limitations and future directions in “Limitations” section. | Section 5.2 (p. 40, lines 776-782)  Section 6 Limitations (p.44-45) |
| 3. Clarity on the process of participants’ recruitment  - Follow the comments of Reviewer 2 regarding this issue by clarifying the participants’ details about their experiences, session times, etc. Explain the consent obtained. Explain how you maintained the validity of the electronic survey. | (1)Clarified recruitment process, consent procedure, and measures for ensuring data validity in the survey. Added detail on preventing duplicate submissions and manual screening.  (2)Participant background was limited to gender, academic title, and teaching experience in the original questionnaire; so we did not provide additional participant details. | Section 3.1 （p.8-9, lines 159-173）  Section 3.5 (p.12) |
| 4. Quantitative Reporting of Qualitative Data  - Please incorporate direct quotes in your qualitative analysis, properly citing participants wherever necessary, in accordance with thematic analysis standards. Update your tables accordingly. For example, there are issues with Table 8 and its interpretations, ensuring all quantitative data in the text aligns with the tables. | (1)Added direct quotes from interviews in qualitative analysis;  (2)Corrected inconsistencies between textual descriptions and Tables 1, 3, 4, and 8, and refined narrative to enhance accuracy. | Section 4.1.4 (p.21, lines 400-401)  Table 1, 3, 4, 7, 8 (p.9, 14, 15, 21, 27, respectively) |
| 5. Discussion needs to be strengthened  - As discussed earlier, your discussion should correlate with your theoretical framework. Various discussed variables are not compared with prior literature, i.e., ‘feedback literacy’ is not discussed compared with the results of Carless (2020), and so on. Moreover, your gaps are also not clear in your discussions | (1)Strengthened the discussion by incorporating relevant literature (e.g., Xu & Brown, 2016; Carless &Winstone, 2023)  (2)Discussed assessment literacy and other key variables in relation to prior studies. | Section 5.1-5.3 (p. 35-44) |
| 6. More Important Suggestions  a. Include a ‘limitations’ section following the ‘discussion’ to address the previously mentioned limitations. | Added a new “Limitations”section (Section 6) addressing geographical and self-reporting limitations. | Section 6 Limitations (p. 44-45) |
| b. Improve clarity and readability by presenting a ‘visual model’ of FA. | A conceptual visual model of formative assessment implementation in the Chinese EFL context has been added, illustrating the interplay between teachers’ beliefs, practices, challenges, and contextual factors. | Fig. 1 at the end of Section 5.3 (p. 43, lines 834-846) |
| c. Importantly, emphasize the ‘policy implications’ within the context of Chinese EFL teachers. | Policy implications have been added and emphasized in the context of Chinese EFL teaching. | Section 5.3 (after conceptual model, p.43-44, lines 847-857) |
|  |  |  |

| **Referee 1’s comments** | Response | Location |
| --- | --- | --- |
| Reviewer #1: The study’s sample is limited to university EFL teachers from a single province in China. While the sample includes a range of institutions and teacher demographics, it does not capture the full diversity of educational settings across China, such as variations in regional educational policies, resource allocation, or institutional cultures. This limits the generalizability of the findings to other provinces or to the national context, where formative assessment practices and challenges may differ significantly due to local factors, differing levels of economic development, or institutional priorities. | Acknowledged the geographical and institutional limitations of the sample in the Discussion section and in the dedicated Limitations section. | Section 5.2 (p. 42, lines 820–823);  Section 6 Limitations (p. 44-45) |
| The reliance on self-reported data through questionnaires and interviews introduces the risk of response bias. Teachers may overstate their use of formative assessment or provide socially desirable responses, especially given the policy emphasis on formative assessment in China. Self-reporting also makes it difficult to verify whether stated practices align with actual classroom behaviors, potentially leading to discrepancies between reported and enacted formative assessment practices. | Acknowledged the potential for response bias due to self-reporting and discussed its implications for data validity. | Section 5.2 (p. 40, lines 776–782);  Section 6 Limitations (p.44-45) |
| The study does not include direct classroom observations or analysis of formative assessment artifacts (such as student work samples, feedback records, or assessment rubrics). Without observational or documentary evidence, it is challenging to fully understand how formative assessment is implemented in practice, the quality of feedback provided, or the real-time challenges teachers face. This methodological limitation restricts the depth and validity of the findings regarding actual classroom practices. This should be discussed at length in the Discussion. | Acknowledged the lack of observational and artifact-based data as a limitation, and suggested incorporating these methods in future research. | Section 6 Limitations (p.45, lines 873-875) |
| **Reviewer #2:** |  |  |
| 1. It is unclear how two researchers managed to recruit and brief 322 participants within 60–90 days. It is recommended that the authors should clarify the recruitment process, including how many conferences were involved and how participants were approached and informed about the study. | Clarified the recruitment process, including the use of snowball sampling and in-person outreach at academic conferences. Details on participant briefing and consent procedures are provided in the Methods section. | Section 3.1 Participants (p.8, lines 159-173);  Section 3.5 Ethical Considerations (p.12, lines 231-241) |
| 2. I could not find page numbering, so I will be referring to the particular pages through page numbers as shown in the PDF file page count. | Page numbers and line numbers have been added to the manuscript to facilitate referencing and improve readability. | Within the text (all pages) |
| 3. On pages 20, 21, and 23 (as per the PDF file), the authors report how many participants defined formative assessment in a particular way. Since qualitative data were thematically analyzed, reporting exact participant counts without context seems inconsistent with qualitative methodology. The authors should clarify their rationale for quantifying these themes or revise this reporting approach. | Revised the reporting of qualitative findings by replacing exact participant counts with qualitative descriptors such as “a few” or “other”, etc. | Page 17, line 323  Page 17, line 329  Page 19, line 370 |
| 4. On page 28(as per the PDF file), the authors state that “36% of teachers report that formative assessment results...” This data point appears to be missing from Table 8. Please include this information in the table or clarify why it was excluded. | (1)Corrected the inconsistency between text and Table 8, where “36%” should be “38%” in the table.  (2)All tables have been carefully checked and updated Tables 1, 3, 4, 7, and 8 to ensure consistency with the textual descriptions. | Table 1, 3, 4, 7, 8 (p.9, 14, 15, 21, 27, respectively) |
| 5. The discussion section currently reads more like an extension of the findings, with heavy use of quantitative data and limited integration of prior research. The authors are encouraged to engage more critically with existing literature, particularly studies on formative assessment in EFL contexts, to strengthen the scholarly grounding of their discussion. | (1)Revised the “Discussion” section to deepen engagement with existing literature on formative assessment in EFL contexts, particularly studies by Carless & Winstone (2020), Xu & Brown (2016), and others.  (2)Reduced quantitative reporting and emphasized interpretation in relation to prior findings. | Sections 5.1–5.3 |
| 6. The manuscript should be revised to fully comply with the Vancouver referencing style, as required by PLOS ONE. This includes proper in-text citations and reference formatting consistent with the journal standards. | The manuscript has been revised to fully conform to the Vancouver referencing style, including in-text citations and reference list formatting. | Entire in-text citations and “References” (p.46-56) |
