# Supplementary material for: Exploring EFL teachers’ beliefs and practices of formative assessment in Chinese context
Source: PLoS One. 2025 Sep 29;20(9):e0333678. doi: 10.1371/journal.pone.0333678 (PMC12478959; doi:10.1371/journal.pone.0333678)
Supplement: Attachment — Submitted filename: Response to Reviewers 20250905.docx [file pone.0333678.s006.docx]

Response letter to editor and reviewers

Dear Editor and Reviewers,

It is our pleasure to submit the revised version of our manuscript, “Exploring EFL Teachers’ Beliefs and Practices of Formative Assessment in the Chinese Context” (PONE-D-25-10820R2), in response to the thoughtful comments from the academic editor and reviewers.

We sincerely appreciate the time and effort from both the editor and the reviewers. We are grateful to Reviewer #1 and Reviewer #2 for confirming that all previous comments have been adequately addressed, and for their positive assessment of the manuscript’s technical soundness, data availability, and clarity.

Below, we provide a point-by-point response to each of the editor’s comments. We have made every effort to address the feedback in a way that aligns with the nature of our study.

Additionally, we have updated the Abstract to more accurately reflect the study’s methodology, specifying that data were collected through surveys and semi-structured interviews.

We hope these responses clarify our approach and demonstrate our commitment to transparency and scholarly rigor. We remain open to any further suggestions that would enhance the clarity and impact of our manuscript.

Sincerely,

Dr. Wanrong Lei

| **Editor’s comments** | **Our Response** | **Locations** |
| --- | --- | --- |
| 1. Lesson Plan Replicability:  - A full transcript may be outlined alongside the lecturer's session, alongside the narrative structure. Including an example lesson plan or structured summary would support replicability. | We appreciate the suggestion.  However, this study did not involve any teaching intervention; it was based on a teacher survey +semi-structured interviews. Therefore, no lesson plans, transcripts, or scripts were generated. | N/A |
| - Similarly, names of the chapters, names of the textbooks, or any other instructional materials may be needed alongside the teacher-led textbook content descriptions. | As above, no specific textbook chapters or instructional materials were used in this study, as it did not involve a teaching intervention. | N/A |
| - The details about the videos, that is included in follow-up classes, should be needed. For instance, who is the creator? What is the script? How is this script relevant? Is the duration of the video is sufficient? What is the source of the video? etc | We thank the editor for this detailed inquiry.  However, no videos were created or used in this study. | N/A |
| 2. Availability of Teaching Materials: - Please provide an English version of the questionnaire, which is a supplement for the international or multidisciplinary audiences. | We appreciate the suggestion. We confirm that the English version questionnaire was originally submitted as “Supporting Information- compressed/ZIP file archive (S1)” in revised submissions. | S1-questionnaire |
| - Are the lectures' notes, scripts, and slides available on request? | No classroom lectures were conducted as part of this study, and therefore no lecture notes, scripts, or slides were produced. | N/A |
| **In summary, areas for improvement are:**  - The description of lesson content, particularly for the teacher-led session, would benefit from the inclusion of exact textbook references, lesson objectives, or example discussion questions.  - For the lecturer-guided session, while the biographical narrative is informative, a more structured or script-based summary (e.g., main talking points, message themes) would be helpful for educators attempting to replicate the intervention. - The video resources used in follow-up classes are not described in sufficient detail (e.g., content summary, creator, duration). - The authors may consider providing an English version of the questionnaire in supplementary material for international audiences. | We thank the editor for these thoughtful suggestions aimed at enhancing replicability and accessibility. We would like to clarify that this research is a survey- and interview-based study exploring teachers’ beliefs and practices of formative assessment, rather than an intervention study. Therefore, no lesson plans, classroom scripts, or multimedia resources were involved.  Regarding the questionnaire, the English version has been available in the submission system as “Supporting Information- compressed/ZIP file archive (S1)” | N/A  S1-questionnaire |
